# Socially assistive robots in child healthcare: evaluating internal and external emotion regulation interventions

**DOI:** 10.3389/frobt.2025.1628795

**Published:** 2025-11-07

**Authors:** Anouk Neerincx, Julian Plat, Maartje M. A. De Graaf

**Affiliations:** 1 Department of Smart Systems for Healthy Living, HU University of Applied Sciences, Utrecht, Netherlands; 2 Department of Information and Computing Sciences, Utrecht University, Utrecht, Netherlands

**Keywords:** child-robot interaction, socially assistive robot (SAR), emotional support, pedriatic healthcare, emotion regulation, vaccination anxiety, active participation, physical comfort

## Abstract

**Introduction:**

Socially assistive robots (SARs) have shown promise in pediatric healthcare by helping children manage the stress and anxiety associated with medical procedures. However, limited research exists on the specific robot behaviors that are most effective in reducing negative emotions in children during stressful interventions. This study aimed to compare the effectiveness of two emotional support strategies provided by a SAR during a vaccination event: internal emotion regulation through a guided breathing exercise and external emotion regulation via motivational speech and physical comfort (hugging). Additionally, we compared the effects of active and passive participation in the two SAR interventions.

**Methods:**

A field study was conducted during annual group vaccination days, involving 225 children aged 8–12 years. Emotional and behavioral outcomes, including anxiety, fear, trust, and willingness to engage with the robot, were measured using self-report questionnaires.

**Results:**

Results indicated that while girls reported higher levels of fear and anxiety than boys, active participation in the SAR intervention led to greater reductions in fear and anxiety, particularly among girls. Additionally, active hugging enhanced both engagement and trust, with girls showing a stronger response to such a physical comfort intervention.

**Discussion:**

These findings indicate that, within the constraints of this study, SAR interventions were associated with reduced negative emotions in children during vaccinations, with active participation and physical comfort being particularly impactful for emotional support. This study offers valuable insights into optimizing SAR interventions in pediatric healthcare.

## 1 Introduction

Socially assistive robots (SARs) have emerged as a promising technology in healthcare (Rabbitt et al., 2015), offering support to clients navigating hospital visits and chronic conditions. Social robots show potential in promoting mental wellbeing (Axelsson et al., 2024). Particularly in child healthcare, SARs harness children’s natural inclination toward interaction and play with robots to provide therapeutic benefits. Recent research on SARs in pediatric settings highlights their potential in helping children regulate negative emotions and stress that often accompany medical treatments. These robots may serve as engaging distractions (Rossi et al., 2020), deliver multi-modal information about hospital environments (Alemi et al., 2014) or medical procedures (Neerincx et al., 2021a; Neerincx et al., 2023a), and offer emotional support through companionship (Jeong et al., 2015; Okita, 2013), motivational speech (Beran et al., 2013), breathing exercises (Matheus et al., 2022), physical comfort such as hugging or petting (Eind and Heerink, 2018), or guided emotion regulation (Alemi et al., 2016; Ali et al., 2021; Looije et al., 2016). Given these advantages, SARs are increasingly integrated into child healthcare applications, including children’s vaccinations (e.g. (Neerincx et al., 2023a; Beran et al., 2013; Rossi et al., 2020)).

Despite the growing interest in social assistive robots (SARs) and their potential benefits, research on their application in pediatric healthcare remains limited and exploratory (Kabacińska et al., 2021; Littler et al., 2021; Moerman et al., 2019). In particular, there is a lack of understanding regarding how different SAR behaviors and interaction modalities can work together to effectively alleviate children’s negative emotions during stressful medical procedures. Additionally, it is unclear how the form of engagement between the child and the robot, whether active or passive, interacts with different SAR behaviors to influence emotional outcomes. The child’s level of participation may shape their effectiveness. Understanding whether emotional regulation benefits depend primarily on the robot’s behavior or on the child’s active involvement is essential for designing adaptive and personalized SAR interventions.

The present study addresses this gap by comparing two emotional support strategies delivered by a SAR: one promoting internal emotion regulation through a guided breathing exercise, and another fostering external regulation via motivational speech and physical comfort. Crucially, we position the child’s participation mode (active vs. passive) as a central variable of interest. We hypothesize that active participation will enhance emotional benefits (reducing fear and anxiety, increasing trust and engagement) relative to passive exposure, and that this effect may interact with the type of support strategy and with individual factors such as gender.

We examine children’s responses to these interventions by assessing anxiety, fear, trust in the robot, and their intention for future use. Additionally, we explore the role of active versus passive participation in shaping these outcomes. By foregrounding participation as a key mechanism of emotional regulation, this study aims to clarify not only what SARs do to comfort children, but also what children do with SARs that makes such comfort effective. Our findings suggest that active participation, especially when involving physical comfort such as hugging, significantly reduces negative emotions and enhances trust. Notably, gender differences emerged, with girls reporting higher baseline anxiety but also demonstrating stronger positive responses to active engagement and physical comfort. These results underscore the value of tailoring SAR behaviors to individual needs and highlight the importance of active engagement strategies in systematically improving emotional support for children undergoing medical procedures.

## 2 Related work

### 2.1 Reducing anxiety in pediatric healthcare settings

Children often experience anxiety and distress during medical procedures, which can lead to negative emotional responses and hinder their cooperation with healthcare providers (Taddio et al., 2022). This is particularly evident in situations involving needles, such as vaccinations, where children may exhibit increased distress and anxiety (Taddio et al., 2022). To address these challenges, various interventions have been developed to help children cope with their emotions in healthcare settings.

For example, parental presence during medical procedures (Hussain and Khan, 2018) and clown therapy (Golan et al., 2009) have both demonstrated some success in alleviating anxiety, though these methods are often limited by practical constraints such as availability and cost (Mathias et al., 2023). Similarly, puppet and therapeutic play interventions, which allow children to engage with and process their medical experiences, have shown potential in lowering anxiety (e.g. (Dehghan et al., 2017)), although the outcome of such interventions are often inconsistent (Mathias et al., 2023). General distraction activities (e.g., toys and games (Aydın et al., 2017)) can also help divert children’s attention away from distressing medical procedures, but their impact tends to be modest (Mathias et al., 2023).

A systematic review on audiovisual interventions for reducing preoperative anxiety (Chow et al., 2016) found that videos, multi-faceted programs, and interactive games are effective strategies, while music therapy and internet-based programs are less effective. These functionalities could be seamlessly implemented in robotic systems.

Despite the diversity of existing interventions studied in pediatric care, there remains a lack of consistency in their overall effectiveness.

Across these interventions, one common factor associated with effectiveness is the child’s degree of active engagement. For instance, therapies that invite children to participate physically or cognitively, such as puppet play, interactive games, or guided coping activities, tend to produce stronger emotional benefits than those relying on passive observation alone (Dehghan et al., 2017; Mathias et al., 2023). However, few studies have explicitly examined how active versus passive forms of engagement contribute to emotional outcomes in medical contexts. This gap is particularly relevant when designing technology-mediated interventions, where interaction dynamics can vary widely between active participation and passive exposure.

### 2.2 Robot-specific interventions

Socially assistive robots (SARs) possess a wide range of capabilities, including supervision, coaching, motivation, and companionship, making them valuable for various populations such as stroke patients and older adults. Children, in particular, are quick to integrate SARs into their treatment routines (Feil-Seifer and Matarić, 2011; Kabacińska et al., 2021). SARs are especially beneficial for children undergoing rehabilitation–a process often marked by difficulty in maintaining motivation. While healthcare workers typically provide motivation, SARs can act as substitutes when professionals are unavailable, encouraging children to complete their exercises by offering demonstrations, verbal feedback, and coaching, which contributes to positive rehabilitation outcomes (McCarthy et al., 2015; Butchart et al., 2021).

The implementation of SARs in child healthcare provides several advantages. First, these robots can present information in a multimodal format (e.g., displaying informative videos while simultaneously engaging in verbal interactions and incorporating nonverbal behavior (Neerincx et al., 2023a)), which can enhance treatment engagement and facilitate more efficient information processing (Sankey et al., 2010). Second, children may find interacting with a robot less intimidating than engaging with adult caregivers (Neerincx et al., 2021b), potentially lowering the barriers to participate in medical treatments and improving compliance. Third, children’s ability to form connections with robots can foster a sense of trust (Van Straten et al., 2020) and familiarity with the medical intervention, contributing to a more positive healthcare experience overall. Last, the technological capabilities of SARs enable personalized care that can be adapted to meet the specific needs and characteristics of the child (e.g., personality, age) (Neerincx and Luijk, 2020).

SARs have been effective within the broader pediatric population in assisting children with autism spectrum disorder (ASD). These robots support social development by teaching and demonstrating socially desirable behaviors, thereby enhancing communication skills and emotional expression for children with ASD (Cho and Ahn, 2016). In child healthcare, SARs have been utilized to help children cope with medical procedures and chronic conditions by leveraging their natural affinity for robots.

SARs can effectively distract children by playing educational videos about the hospital environment (Alemi et al., 2014) or explaining upcoming medical procedures (Neerincx et al., 2021a). Incorporating emotional gestures synchronized with these informative videos has been shown to significantly reduce anxiety levels and increase engagement during the interaction (Neerincx et al., 2023a). While prior research has demonstrated that SARs can inform, distract, or comfort children, little attention has been given to how the child’s level of participation shapes the effectiveness of these robotic interventions. For example, many studies describe interactions where children observe or listen to the robot, but few compare these experiences to active engagement, such as performing guided actions or initiating physical contact.

Several systematic reviews on the application of social robots in pediatric healthcare (Kabacińska et al., 2021; Littler et al., 2021; Moerman et al., 2019) have concluded that such systems show promise in reducing anxiety and distress levels. This suggests that SARs could serve as similarly effective interventions, potentially improving children’s overall wellbeing in healthcare settings.

Similar approaches have been tested in studies using SARs to alleviate negative emotions in child healthcare settings. For instance, a physical robot acting as a play companion has been shown to reduce stress and anxiety more effectively in terms of engaging than a virtual version of the same robot (Jeong et al., 2015). Additionally, robot companions have been successful in decreasing pain and anxiety while simultaneously increasing positive emotions in children (Okita, 2013).

Studies have shown that social robots can be effective in providing distraction during medical procedures, reducing pain and distress. For instance, robots employing distraction strategies during flu vaccinations have been found to reduce children’s pain and discomfort more effectively than traditional care methods (Beran et al., 2013; Rossi et al., 2020). Similarly, robot pets, such as Pleo, have been shown to reduce anxiety by encouraging nurturing behaviors during interactions (Eind and Heerink, 2018).

Building on this evidence, the present study examines not only the type of emotional support provided by a SAR but also how the child’s mode of participation influences emotional outcomes.

### 2.3 Stress management techniques for children in healthcare

Medical procedures, such as vaccinations, often cause significant stress and anxiety for children (Taddio et al., 2022). Various techniques have been developed to help children regulate their emotions and cope with these experiences, which can be broadly classified into internal and external techniques.

Internal techniques focus on teaching children how to manage their emotional responses independently. Simple, age-appropriate breathing techniques can help children regulate their physiological reactions to stress. For instance, mindfulness exercises can reduce anticipatory anxiety by encouraging children to focus on the present moment (Willard, 2006). Psychotherapy sessions incorporating robots have led to significant reductions in anger, anxiety, and depression compared to sessions without robot support (Alemi et al., 2016). In hospital settings, robots like NAO have been used to facilitate self-management for children with chronic conditions, leading to improved mood and increased openness, as reported by parents and hospital staff (Looije et al., 2016). Additionally, SARs that incorporate strategies such as deep breathing and distraction have shown promise in reducing fear and pain during medical procedures (Ali et al., 2021; Farrier et al., 2020).

External techniques, on the other hand, involve interventions provided by external factors, such as caregivers or healthcare professionals. For example, motivational speech, such as compassionate questioning from parents or healthcare providers, can encourage children to feel more in control and help them discover intrinsic motivation (Dacey et al., 2016). Asking questions like “*What would help you feel braver right now?*” empowers children to actively engage in their coping strategies. Nonverbal support, including smiles, comforting touches, or hugs, also provides reassurance and reduces anxiety (Dacey et al., 2016).

Studies have shown that social robots can be effective in providing distraction during medical procedures, reducing pain and distress. For instance, robots employing distraction strategies during flu vaccinations have been found to reduce children’s pain and discomfort more effectively than traditional care methods (Beran et al., 2013; Rossi et al., 2020). Similarly, robot pets, such as Pleo, have been shown to reduce anxiety by encouraging nurturing behaviors during interactions (Eind and Heerink, 2018).

Research indicates that children often initiate physical comfort, such as hugging, with robots during healthcare interactions (Moerman and Jansens, 2021; Logan et al., 2019). While the role of social touch in human-robot interaction is still underexplored (Willemse and Van Erp, 2019), it holds significant potential for future research. In human medical relationships, having a supportive and trusting relationship with a healthcare worker is linked to better treatment outcomes for the patient (Mikesell, 2013). Furthermore, an increasing body of research from behavioral and neural sciences underscores the crucial role that social touch plays in human development and mental wellbeing (Suvilehto et al., 2023). External techniques offered by the robot, such as motivational speech and physical comfort, could therefore potentially increase trust in that robot and enhance the child’s healthcare experience.

### 2.4 Current study

Building on these findings, our study compares the effectiveness of two SAR interventions: (1) promoting internal emotion regulation through a breathing exercise and (2) fostering external emotion regulation through motivational speech and physical comfort.

We operationally define emotion regulation as the processes by which individuals influence which emotions they have, when they have them, and how they experience and express these emotions (Gross, 2015). This definition encompasses both the modulation of emotional intensity (reducing negative emotions like fear and anxiety) and the promotion of positive emotional states that facilitate adaptive responses (such as trust and willingness to engage). Our outcome variables map onto this emotion regulation framework as follows: *Fear and anxiety* represent direct measures of negative emotional state intensity that effective emotion regulation should reduce. *Engagement* (measured through willingness to listen more and meet the robot again) indicates the development of positive emotional associations and adaptive responses to the intervention. *Trust* reflects the establishment of emotional safety and confidence in the robot’s supportive capacity, demonstrating successful emotional support.

Based on the literature, we expect both internal as well as external emotion regulation techniques carried out with a robot to reduce fear and anxiety. However, given the mixed evidence for different emotion regulation approaches in pediatric settings, we pose the following exploratory research questions: (1) Do internal and external emotion regulation interventions differ in their effectiveness for reducing fear and anxiety? (2) Do these interventions differ in their impact on engagement and trust?

Since active participation in mental health and educational interventions has been shown to enhance treatment outcomes and engagement (Jørgensen and Rendtorff, 2018), this study also investigates the role of active versus passive participation in SAR-led emotional support. Prior work suggests that interactive, embodied engagement can strengthen learning and emotional regulation, yet SAR research has rarely isolated participation as an independent factor. In our design, active participation involves children directly engaging with the robot’s prompts (e.g., performing breathing exercises or hugging the robot), while passive participation involves observing or listening without direct action. We hypothesize that active participation will lead to greater reductions in fear and anxiety, and higher engagement and trust, compared to passive participation.

## 3 Methodology

The effectiveness of SAR interventions in child healthcare was explored during a group vaccination event organized annually as part of the Dutch National Immunisation Programme, where a social robot was deployed to inform the children about the procedure by means of a short video developed by the Dutch Child and Family Center.

### 3.1 Participants, research setting and research team

**FIGURE 1:**
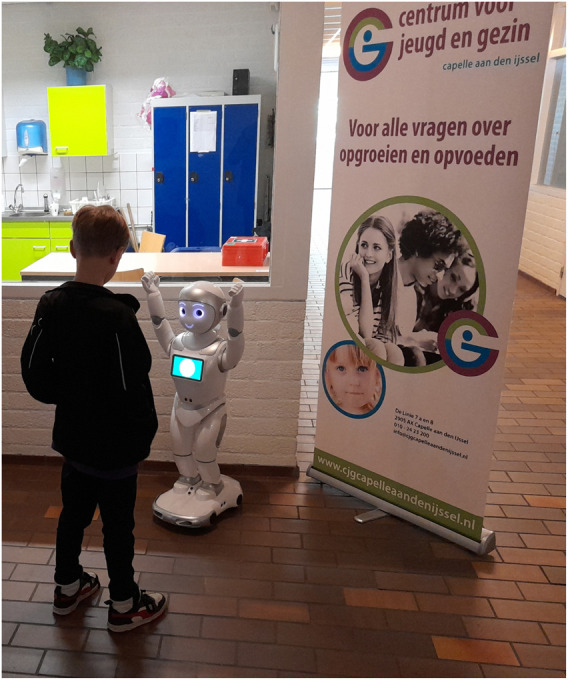
Picture of the study set-up, displaying a child interacting with the robot during the group vaccination day.

Dutch Child and Family Center in Capelle aan de IJssel, the Netherlands, provides general healthcare services to children and their families, including vaccinations, eye tests, check-ups, as well as family coaching and mental health support. Children aged 9 from the surrounding area are invited to vaccinations against HPV and Meningitis as part of the Dutch National Immunisation Programme. Participants were recruited through voluntary sampling. During the two consecutive vaccination days in April 2024, four researchers were present to inform the children and their guardians about the study, activate the robot, and collect questionnaires. We deployed an iPal robot (see Figure 1), as well as a cellphone to control the robot by means of the iRemoter application. The display of the two intervention types were alternated, with the internal emotion regulation intervention in the morning and the external emotion regulation intervention in the afternoon on day one and in reverse order on day two. Due to the layout of the vaccination site, it was not feasible to run both interventions simultaneously nor in random order. However, counterbalancing the morning/afternoon slot over two different days resulted in sufficient randomization. During the interaction, children could freely decide whether to physically or verbally respond to the robot’s prompts. This design enabled us to capture natural variations in participation, distinguishing between active engagement (e.g., performing the breathing exercise or hugging) and passive engagement (e.g., observing without responding).

Participants were recruited through voluntary sampling at the entrance, following an explanation of the study to both the children and their guardians. In total 243 children participated in this study, of which 225 (106 boys, 108 girls, 11 not disclosed) completed the survey (surveys without guardian signature or with >3 missing data points were excluded), with age ranging from 8 to 12 years old (*M* = 8.80, *SD* = 0.85). Ethical approval for the study was granted by the ethics board of Utrecht University.

### 3.2 Study procedure

**FIGURE 2:**
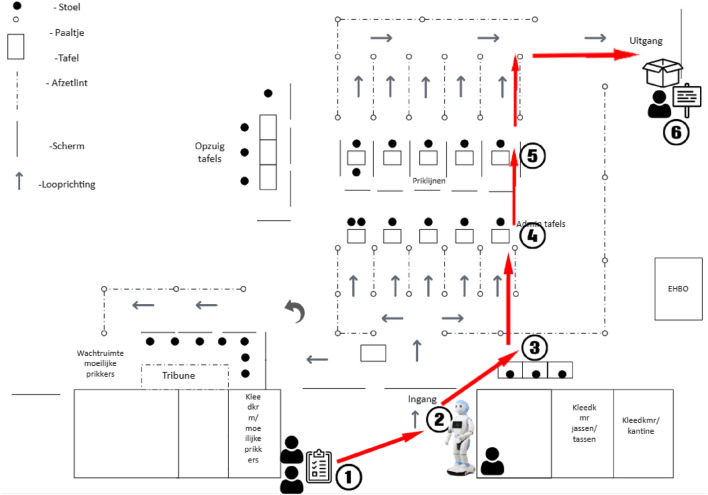
Map of the experiments procedure, with 1. Questionnaire handout, 2. Robot interaction, 3 - 5. Vaccination procedure, 6. Collection of questionnaires.

A schematic overview of the procedure is presented in Figure 2. Upon arrival at the vaccination site, children and their guardians were greeted by one or two researchers, who provided information about the study and asked if they would like to participate. Interested children received a paper questionnaire, and guardians were asked to sign an informed consent form. Children were also informed that they could withdraw from the activity at any time without any consequences. The children were instructed to fill out the first section of the questionnaire while awaiting further instructions, and they were allowed to complete it with their parent or guardian. This section gathered demographic data and assessed the child’s current level of anxiety. The child was then sent to the robot (see location 2 in Figure 2), which displayed one of the two types of intervention. As part of the normal queueing process, the parent or guardian would walk with the child and remain present during the interaction, standing next to or behind the child. Staff remained in their usual locations at least a couple meters away (e.g., the vaccination stations) and were not involved in the child-robot interaction. To preserve the illusion of full autonomy, the SAR operator was seated approximately 2 m away from the robot in a concealed location. The operator’s only involvement was to discreetly trigger the robot’s script through a wireless control interface (on a smartphone) when a child approached from the survey table; from that point onward, the robot operated autonomously. The robot ran a pre-programmed script triggered by the operator, which was the same for all children in that condition. No spontaneous dialogue occurred; all interactions followed predetermined sequences and no unscripted exchanges occurred. After the SAR intervention (although the children were free to leave at any point), the children continued through the queue for registration and vaccination (see location 3 in Figure 2). Following vaccination, the participants headed towards the exit, where they were asked to complete the second part of the questionnaire (including a measurement for behavioral compliance (i.e., active participation) during the SAR intervention). This marked the end of the study and participants were asked to return their completed questionnaire and thanked for their participation.

### 3.3 SAR intervention types

During the study procedure, two distinct SAR interventions were implemented, while the children would only be exposed to one of these. The scripts for both interventions were brief and approximately equal in duration (i.e., 40 s, followed by a video of 1 min). Both interactions started with the robot giving a brief introduction (“*Hello, my name is Chris. It’s nice to meet you.*”). The first SAR intervention condition aimed at promoting *internal* emotion regulation through a *breathing* exercise, while the second SAR intervention fostered *external* emotion regulation through *motivational speech* and *hugging* as physical comfort. The second part of the intervention was similar in both conditions, with the robot showing a short informational video about vaccinations and concluding with a goodbye tailored to the respective condition. The informative video was started post-intervention to maintain the child’s engagement during the interaction and prepare for the vaccination.

#### 3.3.1 Breathing condition

In the breathing condition, the robot would introduce the breathing exercise by stating the following: “*There are certain ways that we can breathe to help us relax when we are feeling nervous–we can do something called ‘belly breathing.’ It’s called belly breathing because you breathe so deeply that you can feel it in your belly. Put your hand on your belly so that you can feel when the air goes in and out of your body. Breathe in slowly through your nose and feel your belly expand. Now breathe out slowly through your mouth, and feel your belly go back in. Say ‘haa’ as you breathe out. Try to keep your shoulders relaxed too.*” After the informational video about vaccinations was played on the robot, the robot concluded the interaction with a deliberately neutral encouraging statement to keep the focus on internal emotion regulation processes: “*Good luck with the vaccination.*”

#### 3.3.2 Hugging condition

In the hugging condition, the robot gave a short motivational speech and offered a hug as follows: “*I want you to feel comfortable during receiving the vaccine. When you get to the vaccine location, you can tell the doctor which arm you prefer. You should also say something if you’re not comfortable. If you think it will make you feel better, you may give me a hug.*” After this prompt, the robot widened its arms while remaining stationary for 5 s, before showing the informational video about vaccinations. The interaction concluded with an explicitly motivational goodbye designed to provide external encouragement: “*Good luck with the vaccination, I know you can do it!*”

Although the two SAR interventions differed in their emotional regulation strategies, i.e., internal (breathing) versus external (hugging and motivational speech), each also afforded varying degrees of child participation. The breathing exercise invited children to perform a guided physical activity, while the hugging condition encouraged embodied interaction through tactile engagement. However, in both conditions, participation was voluntary, allowing us to compare the emotional impact of active versus passive engagement across both regulatory strategies.

Participant gender was equally distributed between experimental conditions ( $\chi^{2}\left(1\right)=0.66$ , p=.416 ), with a total of 101 children (53 boys, 48 girls) in the breathing condition and 113 children (53 boys, 60 girls) in the hugging condition. Table 1 shows an overview of final participant numbers per variable^1^. A Chi-square test was insignificant, indicating equal distribution over different conditions.

**TABLE 1:** The number of participants per SAR intervention type, gender, and observed intervention participation during the interaction.

Intervention	Gender	Participation			Total
		Passive	Active	Unknown	
Breathing	Boys	13	17	23	53
	Girls	15	14	19	48
	Unknown	0	1	5	6
Hugging	Boys	14	26	13	53
	Girls	18	29	13	60
	Unknown	0	2	3	5
	Total	60 (26.7%)	89 (39.6%)	76 (33.7%)	225 (100%)

### 3.4 Measurements and data analysis

**FIGURE 3:**
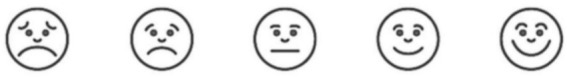
The smileys used in the survey for all 5-point scale questions (engagement, fear, trust, and anxiety). Based on the question-specific corresponding answer options, the smileys were accompanied by text below (e.g., “*not at all, probably not, not sure, probably yes, definitely*”).

An overview of our measurements, collected through self-report questionnaires before and after the SAR intervention, is shown in Table 2. First we requested children’s *intervention participation* with two options: (1) active (i.e., gave the robot a hug/performed the breathing exercise), (2) passive (i.e., only observed the robot trying to hug/showing the breathing exercise). Engagement, fear and trust were assessed using a questionnaire developed by Looije et al. (Looije et al., 2008). To evaluate *Engagement*, we asked if the children would like to meet the robot again, and listen to the robot for longer, indicating potential future interaction and sustained engagement. *Fear* was measured by asking children how they felt about receiving the vaccine both before and after the vaccination. *Trus*t was gauged by asking whether they believed the robot was telling the truth. Additionally, *Anxiety*, a time-limited emotional state (Russell and Barrett, 1999), was measured using a question adapted from the Modified Short State-Trait Anxiety Inventory for Children (Nilsson et al., 2012; Spielberger, 1970): “In this moment, I feel calm.” This item was administered before and after the vaccination and utilized similar facial expressions to represent emotions. All these self-report questions were presented on a 5-point scale ranging from 1 (Surely not) to 5 (Yes, very much), with corresponding smiley faces, a method recommended for self-reports with children (Read et al., 2002). The scale with smiley faces used in the questionnaire is displayed in Figure 3. Fear and Anxiety were measured pre- and post, to capture potential benefits of the SAR intervention on fear and anxiety levels. Engagement and Trust were measured after the interaction took place.

**TABLE 2:** Summary of measurements, corresponding questions, and answer types, derived from self-report questionnaires.

Measurement	Question	Answer type
Active/passive participation	Gave the robot a hug/performed the breathing exercise, or only observed the robot?	Binary
Engagement (future interaction) (post)	Would you like to meet the robot again?	5-point scale
Engagement (sustained) (post)	Would you like to listen to the robot for longer?	5-point scale
Fear (pre- and post)	How do you feel about receiving the vaccine?	5-point scale
Trust (post)	Do you believe the robot is telling the truth?	5-point scale
Anxiety (pre- and post)	In this moment, I feel calm	5-point scale

## 4 Results

To explore observed emotional outcomes across SAR intervention types and children’s participation levels in child healthcare, we ran several ANOVAs with SAR intervention type (breathing vs. hugging), participant gender (boys vs. girls), and intervention participation^2^ (active vs. passive) as independent between-subjects variables. Box’s Test of Equal Covariance, and Levene’s Test of Equality of Error variances showed normality of the data, except for meet again and trust. We used the Sums of Squares Type 3 model for ANOVA robust to unequal sample sizes and reported estimated marginal means. For an overview of participant distribution, see Table 1. For an overview of all ANOVA results, see Table 3.

**TABLE 3:** ANOVA results for observed differences across SAR intervention types, gender, and intervention participation on fear, anxiety, engagement, and trust.

Dependent and independent variables	F	p	$\eta^{2}$
Fear			
Pre-Post Fear	0.49	0.487	0.00
Pre-Post Fear × Intervention Type	0.20	0.658	0.00
Pre-Post Fear × Gender	0.07	0.787	0.00
Pre-Post Fear × Intervention Participation	5.08	**0.026**	0.04
Pre-Post Fear × Intervention Type × Gender	0.50	0.481	0.00
Pre-Post Fear × Intervention Type × Intervention Participation	0.26	0.611	0.00
Pre-Post Fear × Gender × Intervention Participation	0.30	0.583	0.00
Pre-Post Fear × Intervention Type × Gender × Intervention Participation	0.52	0.473	0.00
Anxiety			
Pre-Post Anxiety	8.75	**0.004**	0.06
Pre-Post Anxiety × Intervention Type	1.03	0.312	0.01
Pre-Post Anxiety × Gender	1.17	0.281	0.01
Pre-Post Anxiety × Intervention Participation	4.15	**0.044**	0.03
Pre-Post Anxiety × Intervention Type × Gender	0.04	0.848	0.00
Pre-Post Anxiety × Intervention Type × Intervention Participation	0.03	0.873	0.00
Pre-Post Anxiety × Gender × Intervention Participation	4.64	**0.033**	0.06
Pre-Post Anxiety × Intervention Type × Gender × Intervention Participation	0.05	0.817	0.00
Engagement - Meet Again			
Intervention Type	1.31	0.255	0.01
Gender	1.62	0.206	0.01
Intervention Participation	16.05	**0.000**	0.10
Intervention Type × Gender	0.22	0.643	0.00
Intervention Type × Intervention Participation	1.98	0.161	0.01
Gender × Intervention Participation	0.20	0.655	0.00
Intervention Type × Gender × Intervention Participation	4.59	**0.034**	0.03
Engagement - Listen More			
Intervention Type	0.00	0.999	0.00
Gender	0.10	0.750	0.00
Intervention Participation	7.64	**0.007**	0.05
Intervention Type × Gender	0.12	0.725	0.00
Intervention Type × Intervention Participation	1.26	0.263	0.01
Gender × Intervention Participation	0.88	0.350	0.01
Intervention Type × Gender × Intervention Participation	4.22	**0.042**	0.03
Trust			
Intervention Type	0.30	0.585	0.00
Gender	0.11	0.744	0.00
Intervention Participation	4.20	**0.042**	0.03
Intervention Type × Gender	0.01	0.946	0.00
Intervention Type × Intervention Participation	5.74	**0.018**	0.04
Gender × Intervention Participation	0.63	0.430	0.01
Intervention Type × Gender × Intervention Participation	0.14	0.714	0.00

Bold values denote statistical significance at p < 0.05.

### 4.1 Fear

**FIGURE 4:**
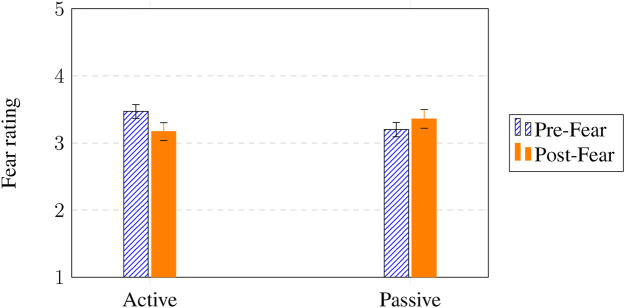
Pre- and post fear × intervention participation (active vs. passive). Y-axis shows the mean values on the 5-point Likert scale used (see Figure 3).

A mixed-design split-plot ANOVA for fear revealed a significant interaction effect for pre and post fear × intervention participation ( F(1,138)=5.08 , p=.026 , $\eta^{2}=.04$ ). Figure 4 shows that active participation (pre fear *M* = 3.47, post fear *M* = 3.17) was associated with a greater decline in fear after interaction with the robot compared to passive participation (pre fear *M* = 3.20, post fear *M* = 3.36), independent of SAR intervention. Fear tended to drop only for those who participated, so the change in fear from before to after the session differed depending on whether the participant *actively took part* in the SAR intervention, with a small-to-moderate effect size. This pattern suggests that children’s emotional improvement depended more on their level of engagement with the robot than on which emotional regulation strategy was offered. Active participation thus appears to be a critical mechanism through which SARs influence children’s affective states.

We also observed a main general effect for gender ( F(1,138)=7.52 , p=.007 , $\eta^{2}=.05$ ) with girls reporting higher overall fear (*M* = 2.60) than boys (*M* = 2.08).

All other main and interaction effects were insignificant.

### 4.2 Anxiety

Another mixed-design split-plot ANOVA for anxiety revealed that anxiety decreased overall from pre-to post-test, with a medium effect size ( F(1,138)=8.75 , p=.004 , $\eta^{2}=.06$ ). More importantly, we found a significant interaction effect for pre and post state anxiety × intervention participation ( F(1,138)=4.15 , p=.044 , $\eta^{2}=.03$ ), telling us that the size of that decrease depends on whether the participant *actively took* part in the SAR intervention. We also found a significant interaction effect for pre and post state anxiety × gender × intervention participation ( F(1,138)=4.64 , p=.033 , $\eta^{2}=.06$ ), which tells us that the participation effect itself was different for boys and girls (e.g., girls who actively participated may have shown a larger decrease in anxiety). In short, anxiety fell overall, but the size of that fall depended on whether the participant actively took part in the SAR intervention, and that participation effect itself varied by gender.

**FIGURE 5:**
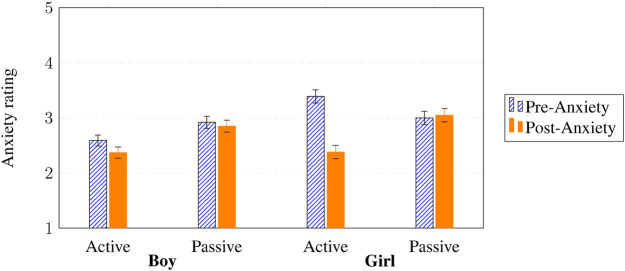
Pre- and post state anxiety × gender (boy vs. girl) × intervention participation (active vs. passive). Y-axis shows the mean values on the 5-point Likert scale used (see Figure 3).

Figure 5 shows that active participation (pre state anxiety *M* = 2.99, post state anxiety *M* = 2.37) resulted in a greater decline of anxiety compared to passive participation (pre state anxiety *M* = 2.92, post state anxiety *M* = 2.85), and this effect for active participation was even larger for girls (pre state anxiety *M* = 3.39, post state anxiety *M* = 2.38) compared to boys (pre state anxiety *M* = 2.59, post state anxiety *M* = 2.37), independent of SAR intervention. This again highlights that participation, not intervention type, explained the strongest reductions in anxiety, suggesting that emotional regulation in SAR contexts may be driven by engagement intensity rather than the specific technique applied.

We also observed a main general effect for gender ( F(1,138)=9.36 , p=.003 , $\eta^{2}=.06$ ) with girls reporting higher state anxiety (*M* = 3.01) than boys (*M* = 2.58). All other main and interaction effects were insignificant.

### 4.3 Engagement

**FIGURE 6:**
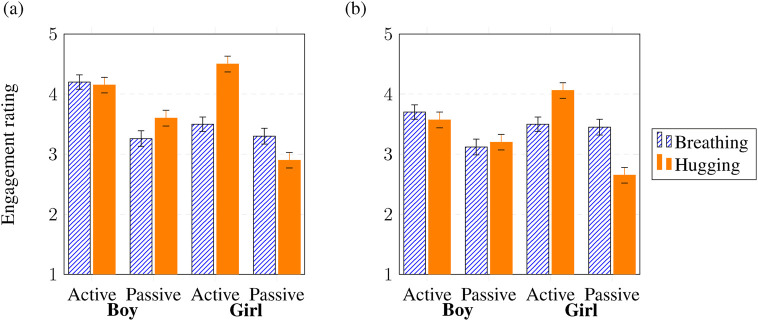
Interaction effects of SAR intervention (breathing vs. hugging) × gender (boy vs. girl) × intervention participation (active vs. passive) on re-engagement. Y-axis shows the mean values on the 5-point Likert scale used (see Figure 3). **(a)** Willingness to meet the robot again. **(b)** Willingness to listen to the robot for longer.

A 3-way ANOVA revealed a significant interaction effect for SAR intervention type × gender × intervention participation on willingness to meet the robot again ( F(1,138)=4.59 , p=.034 , $\eta^{2}=.03$ ) and a main effect intervention participation ( F(1,138)=16.05 , p<.001 , $\eta^{2}=.10$ ). Figure 6a shows that overall active (*M* = 4.07, vs. passive *M* = 3.26) participation resulted in higher willingness to meet the robot again, while active hugging was most effective for girls’ willingness to meet the robot again (*M* = 4.50, vs. boys *M* = 4.14).

Another 3-way ANOVA revealed a similar significant interaction effect for SAR intervention type × gender × intervention participation on willingness to listen to the robot for longer ( F(1,138)=3.97 , p=.050 , $\eta^{2}=.05$ ) and a main effect Intervention Participation ( F(1,138)=9.04 , p=.004 , $\eta^{2}=.10$ ). Figure 6b shows that overall, active participation (*M* = 3.70, vs. passive *M* = 3.12) was associated with higher willingness to listen to the robot for longer. In particular, active hugging was associated with a higher reported willingness among girls (*M* = 4.06) compared to boys (*M* = 3.57).

This supports the notion that participation itself fosters positive relational outcomes, as children who physically engaged with the robot not only reported lower fear and anxiety but also expressed a stronger desire to re-engage.

### 4.4 Trust

**FIGURE 7:**
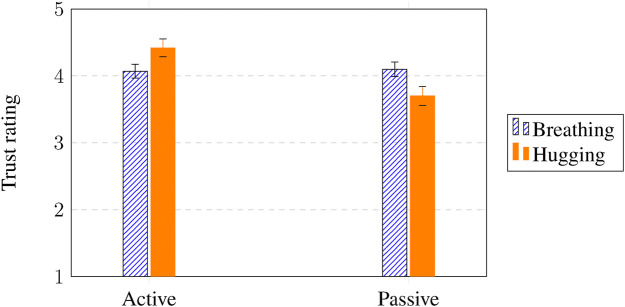
Interaction effects of SAR intervention (breathing vs. hugging) × intervention participation (active vs. passive) on trust. Y-axis shows the mean values on the 5-point Likert scale used (see Figure 3).

A 3-way ANOVA revealed a significant interaction effect for SAR intervention and intervention participation on trust ( F(1,143)=5.74 , p=.018 , $\eta^{2}=.04$ ) and a significant main effect for intervention participation ( F(1,143)=4.20 , p=.042 , $\eta^{2}=.03$ ). Figure 7 shows that active (*M* = 4.25, vs. passive *M* = 3.87) participation was associated with higher trust in the robot, especially actively hugging (*M* = 4.42, vs. active breathing *M* = 4.07) the robot resulted in highest trust. Trust therefore seems to be built not simply through exposure to supportive behaviors, but through mutual interaction: the child’s own involvement reinforcing perceptions of the robot as responsive and caring.

## 5 General discussion

The goal of this research was to examine how children’s level of participation (active vs. passive engagement) influences the effectiveness of two emotional support strategies delivered by a Socially Assistive Robot (SAR) to reduce anxiety and fear in children during vaccination. Specifically, we compared a breathing exercise (internal emotion regulation) and motivational speech combined with hugging (external emotion regulation) during a real-world pediatric vaccination event. A field study was conducted during annual vaccination days, where different SAR versions were deployed, and key outcomes in terms of anxiety, fear, trust, and willingness to engage with the robot were measured.

### 5.1 Fear and anxiety

Our results demonstrate that the child’s mode of participation was a key determinant of emotional outcomes. Active participation led to greater reductions in fear and anxiety, particularly among girls, independent of the SAR’s regulatory strategy. This result aligns with prior systematic findings indicating that robot interventions can alleviate negative emotions in pediatric healthcare (Kabacińska et al., 2021; Littler et al., 2021; Moerman et al., 2019). Our study extends this evidence by identifying the role of active participation as a key factor that may explain inconsistencies observed in earlier research. Specifically, the effectiveness of SAR interventions appears depend on the degree to which children actively engage in the interaction, echoing research showing that physical robots tend to sustain engagement more effectively than virtual counterparts (Jeong et al., 2015).

Additionally, we observe no significant differences between external (motivational speech and physical comfort) and internal (breathing exercises) regulation techniques. This suggests that, within the constraints of our study, both strategies are equally effective in the short-term, potentially because they build on established, non-robotic methods for stress management in children (e.g. (Beran et al., 2013; Rossi et al., 2020; Ali et al., 2021; Farrier et al., 2020)). This finding contrasts with some studies reporting variability in traditional intervention outcomes (Mathias et al., 2023), emphasizing the need for future research to identify moderating factors (e.g., age, gender, context) in the effectiveness of SAR interventions. The comparable effects of internal and external strategies suggest that what matters most is not how the robot supports the child, but how the child participates in that support. This reframing positions participation as a mediator between SAR design and emotional benefit.

### 5.2 Engagement

Active participation also increased children’s willingness to re-engage with the robot, independent of SAR intervention type. This effect was especially strong among girls, from whom the active hugging intervention yielded the highest levels of willingness to engage with the robot again. Prior CRI research suggests that girls place greater emphasis on emotional intimacy and alliance in friendships, which may account for their stronger preference for socially expressive robot behaviors (Westlund et al., 2018). Similarly, studies on affective touch indicate that women more often initiate and evaluate touch as more pleasant than men (Stier and Hall, 1984; Russo et al., 2020), suggesting that incorporating physical comfort in SARs could be particularly engaging for girls.

This finding aligns with studies showing that physical robots acting as play companions can elicit higher engagement than virtual versions (Jeong et al., 2015) or non-interactive alternatives (Aydın et al., 2017). Given that engagement is a central component of therapeutic effectiveness, future research should explore how to optimize SAR interventions to appeal both boys and girls, potentially tailoring the type of emotional support provided to individual preferences and needs (Neerincx and Luijk, 2020).

Beyond the gender effects observed in this study, other child characteristics may also influence participation levels. For instance, personality traits such as extroversion might affect how readily children initiate or sustain active engagement with the robot (e.g. (Neerincx et al., 2023b)). In previous research, it was suggested that anxiety levels also influence whether children will participate in SAR interventions or not (Neerincx et al., 2023a). Future research incorporating richer behavioral measures, such as personality tests, video-based interaction coding or motion tracking, could help clarify how these individual differences shape both the degree and quality of participation. Such insights would inform the design of adaptive SARs that tailor engagement strategies to each child’s disposition and comfort level.

Together, these results underscore that engagement is not just an outcome but also a mechanism of therapeutic benefit: children who engage actively with the robot appear to derive both emotional relief and stronger affiliative motivation.

### 5.3 Trust

Children who actively participated in the SAR interventions reported higher levels of trust in the robot, with the active hugging intervention generating the highest trust ratings. This finding is consistent with previous studies showing that supportive, trusting relationships foster stronger therapeutic alliance and better healthcare outcomes (Mikesell, 2013). The finding that active participation increased trust reinforces the broader pattern observed across all dependent measures: trust, emotional relief, and re-engagement are all shaped by how children participate with the robot, not only by what the robot does.

Physical interaction, particularly hugging, appears particularly influential in fostering trust, potentially due to the central role of social touch in human development and mental wellbeing (Suvilehto et al., 2023). Our results support previous research suggesting that incorporating social touch into SAR interventions can strengthen children’s sense of support during medical procedures (Logan et al., 2019; Moerman and Jansens, 2021). Another noteworthy factor is children’s perception of the robot “breathing.” This subtle cue may have enhanced anthropomorphism by conveying a sense of aliveness, which in turn strengthened trust. Prior work suggests that anthropomorphic features increases trust in social robots (Natarajan and Gombolay, 2020). Since trust is closely tied to both robot acceptance (Song et al., 2024) and therapeutic effectiveness (Hall et al., 2001), further research should investigate how SARs can adapt social touch and other external regulation techniques to suite the emotional and developmental needs of children in healthcare contexts.

While our findings highlight the potential of physical comfort through robot-mediated touch, it is important to distinguish between robotic and human touch. Specific controlled human-robot comparisons are needed to further understand these differences. Human touch conveys complex affective, physiological, and social cues that robots cannot fully replicate (Willemse and Van Erp, 2019). The tactile interaction with a SAR is thus better understood as a symbolic or supportive gesture rather than an equivalent to human caregiving touch. Future research should further examine how children perceive robotic touch, and how such touch can be designed to complement, rather than replace, human emotional support during medical procedures.

### 5.4 Personalization of SAR interventions

Our findings suggest that optimizing SAR interventions requires designs that invite and sustain active participation. Encouraging children to mirror the robot’s breathing or initiate physical comfort may strengthen emotional regulation and trust. Additionally, the robot might offer different emotion regulation techniques based on the child’s preferences, such as offering a choice between breathing exercises or hugging. Robots could also adapt their behavior dynamically to the child’s emotional state. For instance, offering a comforting hug or engage in a breathing exercise when a child appears visibly anxious. This adaptive approach aligns with the principles of personalized medicine and has been shown to enhance the effectiveness of interventions across healthcare contexts (Goetz and Schork, 2018). Moreover, adaptive SARs may enhance acceptance (Mazuz and Yamazaki, 2024).

Gender preferences should be taken into account. Designing social robot embodiments that invite comforting touch (e.g., through soft huggable exterior) may be particularly effective in reducing negative emotions among girls. Our results further identifies gender differences in baseline anxiety and fear levels, with girls reporting higher levels of distress during medical procedures compared to boys. This difference underscores the importance of considering gender-specific emotional needs when designing SAR interventions. Prior research has shown that girls place grater value on emotionally supportive interaction (Westlund et al., 2018), which may explain the strong effectiveness of social touch (i.e.,hugging) intervention for reducing negative emotions among girls.

### 5.5 Human-led versus SAR emotional support

While SAR-led emotional support can reduce anxiety, increase engagement, and foster trust during pediatric procedures, it should be understood in the context of existing human-led interventions. Human caregivers provide rich empathy, nuanced nonverbal communication, and real-time adaptation to a child’s emotional state, qualities that current robots cannot fully replicate yet (Mikesell, 2013; Suvilehto et al., 2023). Techniques such as parental presence, motivational speech, therapeutic play, and clown therapy have demonstrated the importance of individualized and responsive emotional support, though they are often constrained by availability, cost, or situational logistics (Hussain and Khan, 2018; Golan et al., 2009; Dehghan et al., 2017; Mathias et al., 2023).

SARs, by contrast, offer consistency, accessibility, and novel engagement opportunities: they can deliver distraction, guidance, or comforting gestures repeatedly and reliably, and can adapt their multimodal behaviors to the child’s observable engagement (Neerincx et al., 2023a; Beran et al., 2013; Rossi et al., 2020). Importantly, while SARs can emulate some aspects of human-led support–such as motivational prompts or gentle physical cues–they do not replicate the depth and immediacy of human empathy. Instead, these robots are best conceptualized as complementary tools that can augment care, particularly in situations where human resources are limited or children face procedural anxiety without immediate caregiver support.

Future research should compare SAR-led and human-led interventions directly, examining differences in immediacy, emotional depth, and longer-term effects on coping, engagement, and trust. Such studies could clarify how SARs can be integrated into pediatric healthcare to enhance, rather than replace, the emotional support provided by human caregivers.

### 5.6 Limitations and future research

Our results show that SAR interventions can effectively reduce anxiety and fear in children during vaccinations, particularly when children actively participate in the intervention. However, several limitations should be considered when interpreting these findings. First, general vaccination effects might have contributed to the observed reductions in anxiety and fear. Anticipation and subsequent relief are inherent to the vaccination event itself and may have influenced children’s responses independent of the SAR intervention. Nonetheless, our data show that active participation in the SAR intervention was associated with greater reductions in anxiety and fear, suggesting a unique contribution of the SAR intervention to emotional regulation beyond the general vaccination experience.

Second, while our study distinguished between SAR intervention participation levels, the absence of a control group limits direct comparisons with traditional interventions. Prior research has established the general benefits of SARs in medical settings compared to control conditions (Alemi et al., 2016; Ali et al., 2021; Beran et al., 2013; Neerincx et al., 2021a), but further targeted studies are needed to examine how specific emotion regulation strategies, such as external (e.g., physical comfort) versus internal (e.g., breathing), are differentially supported by SARs. Such research would help clarify the mechanisms through which SARs contribute to emotional wellbeing, beyond their established overall utility. Relatedly, a digital placebo effect warrants consideration. Children’s expectations, the novelty of the robot, or its perceived technological sophistication themselves may contribute to positive outcomes, independent of the actual intervention design. Accounting for such effects in future research could disentangle genuine therapeutic mechanisms from expectancy-driven improvements. Exploring mixed interventions that combine both internal and external regulation techniques could reveal whether these approaches produce additive benefits.

Third, the optimization of SAR interventions require some form of adaptive AI modeling, such as rule-based tuning or reinforcement learning guided by a clearly defined optimization criterion. This was beyond the scope of our exploratory study. Future research should first identify such criteria (e.g., minimizing anxiety, maximizing engagement, enhancing trust) and then implement appropriate modeling methods in subsequent iterations of the SAR. A systematic, evidence-based approach will allow SAR behavior to be tailored dynamically to children’s individual needs in real-time.

Fourth, our study focused on two emotion regulation approaches (internal breathing-based and external comfort-based interventions), yet effective support may require integration of a broader set of complementary strategies. Future research should explore additional modalities such as visual empathy cues, adaptive timing based on individual child responses, personalized dialogue systems, and multi-sensory feedback. Integrating multiple strategies might yield stronger and more holistic therapeutic benefits than individual strategies alone.

A fifth limitation concerns the lack of direct evidence for the underlying psychological of physiological mechanisms of the interventions. We did not measure whether breathing prompts activated parasympathetic response or whether hugs worked via social-support appraisals. Without identifying such mechanisms, it is difficult to generalize the findings or explain why certain outcomes, such as fear, anxiety, and trust, were most affected. Future studies should therefore incorporate physiological or psychological measures to clarify these pathways.

Sixth, our study was also limited in scope. It was conducted in the Netherlands, with one robot type, and a specific experimental setting. Cultural factors may shape how children respond to robots and physical comfort, making cross-cultural replication essential to establish broader generalizability. Age-related differences also deserve further attention. Our sample (ages 9–12) spans an important developmental transitions, but our study was not powered to detect developmental effects. Moreover, the presence of care staff, though remaining at their usual stations (approximately 3–4 m away) per standard practice and not engaging with children during SAR intervention, may have influenced children’s experiences. The group setting also allowed children to observe the robot before their own interaction, potentially shaping their expectations. While this introduces bias, this dynamic reflects real-world healthcare conditions, where children naturally witness and learn from others, and thus enhances the ecological validity of the study.

Some practical constraints further limited our study. We deployed the robot available within the Dutch Child and Family Center, which featured a synthetic-sounding voice that may have impacted levels of trust. Future research should employ robots with more natural vocal capabilities. Interaction time was also restricted to approximately 2 minutes, with only 40 s of active intervention. This brevity may have constrained the potential impact of the SAR. Additionally, the robot occasionally struggled to sustain children’s attention for the complete duration of the intervention. Shorter or more engaging interaction designs may yield stronger intervention outcomes.

Lastly, the real-world setting contributed to incomplete and mistimed questionnaire responses. Missing data were notable for both gender (*n* = 11) and intervention participation (33%; *n* = 76). Missing data in this setting might have occurred in general due to the vaccination flow and potential rush of the parents. The high amount of missing data specifically on the intervention participation question may have been due to the absence of a smiley scale on that item, making it visually inconsistent with the rest of the questionnaire and potentially overlooked by participants. This limits the generalization of our results. Future studies should aim for greater control over administration and ensure consistent visual design to maintain attention and data quality.

## 6 Conclusion

This study examined how socially assistive robots (SARs) can support children’s emotional wellbeing during vaccination procedures by comparing two emotion regulation strategies, namely, guided breathing (internal regulation) and motivational speech with hugging (external regulation). Beyond the type of intervention, our findings highlight the critical role of active participation in shaping children’s emotional and relational outcomes. Across both intervention types, children who actively participated (e.g., by hugging or participating in the breathing exercise) reported greater reductions in fear and anxiety, higher trust, and stronger willingness to re-engage with the robot.

These results suggest that the effectiveness of SAR interventions depends not merely on the emotional content or modality of support, but on how actively children are involved in the interaction. Active engagement likely enhances feelings of agency and social reciprocity, strengthening both emotional regulation and trust in the robot. This may help explain the inconsistent effects observed in previous SAR studies that did not distinguish between active and passive user roles.

Moreover, the findings reveal gender differences: girls reported higher baseline anxiety but also benefited more from active, socially expressive interventions such as hugging. This suggests that physical and emotionally supportive engagement may be particularly effective for girls, underscoring the importance of designing personalized SAR experiences that match children’s preferences and emotional needs.

Overall, our study advances the understanding of SARs in pediatric care by identifying participation as a key determinant of emotional effectiveness. Designing robot interactions that invite or scaffold active participation, such as shared breathing, interactive gestures, or child-led touch, may significantly enhance the therapeutic impact of SARs. Future research should explore adaptive systems that dynamically adjust the level of participation to each child’s comfort and engagement level. By doing so, SARs can move beyond passive distraction tools to become active partners in children’s emotion regulation and healthcare experiences.

## Data Availability

The datasets presented in this article are not readily available because we collected data of vulnerable participants and decided to not make it public. Requests to access the datasets should be directed to m.m.a.degraaf@uu.nl.

## Footnotes

1 The participation variable was determined based on observed behavior during the interaction, and not a pre-determined condition.

2 Due to missing data (*n* = 76) for this item, ANOVAs including this dependent variable was run on a sample of *n* = 149.
